# Events Associated with Early Age-Related Decline in Adventitious Rooting Competence of *Eucalyptus globulus* Labill

**DOI:** 10.3389/fpls.2017.01734

**Published:** 2017-10-10

**Authors:** Márcio L. Aumond, Artur T. de Araujo, Camila F. de Oliveira Junkes, Márcia R. de Almeida, Hélio N. Matsuura, Fernanda de Costa, Arthur G. Fett-Neto

**Affiliations:** ^1^Plant Physiology Laboratory, Center for Biotechnology and Department of Botany, Federal University of Rio Grande do Sul, Porto Alegre, Brazil; ^2^Graduate Program in Cellular and Molecular Biology, Center for Biotechnology, Federal University of Rio Grande do Sul, Porto Alegre, Brazil

**Keywords:** adventitious rooting, *Eucalyptus*, juvenility, auxin, gene expression

## Abstract

The development of adventitious roots is affected by several factors, including the age of the cutting donor plant, which negatively affects rooting capacity. *Eucalyptus globulus* quickly loses rooting capacity of cuttings as the donor plant ages, although the molecular and biochemical mechanisms behind this process are still unclear. To better understand the bases of rooting competence loss in *E. globulus*, the time required for a significant decline in rhizogenic ability without exogenous auxin was determined in microcuttings derived from donor plants of different ages after sowing. Tip cuttings of donor plants were severed before and after loss of rooting competence of microcuttings to test the hypothesis that auxin and carbohydrate homeostasis regulate rooting competence decline. There were no significant changes in concentration of carbohydrates, flavonoids, or proteins before and after the loss of rooting capacity. Peroxidase (EC 1.11.1.7) total activity increased with loss of rooting competence. Auxin concentration showed the opposite pattern. In good agreement, *TAA1*, a key gene in auxin biosynthesis, had lower expression after loss of rooting capacity. The same applied to the auxin receptor gene *TIR1*, suggesting reduced auxin sensitivity. On the other hand, genes associated with auxin response repression (*TPL*, *IAA12*) or with the action of cytokinins, the rhizogenesis inhibitor-related *ARR1*, showed higher expression in plants with lower rooting competence. Taken together, data suggest that age negatively affects *E. globulus* rooting by a combination of factors. Decreased endogenous auxin concentration, possibly caused by less biosynthesis, lower auxin sensitivity, higher expression of genes inhibiting auxin action, as well as of genes related to the action of cytokinins, appear to play roles in this process.

## Introduction

*Eucalyptus globulus* Labill. is considered one of the top species for the paper industry due to its high quality cellulose pulp, low lignin and lipid content, and high syringyl/guaiacyl (S/G) ratio (Cruz et al., 2006; Rencoret et al., 2007; Barbosa et al., 2008; Neiva et al., 2014). However, this is a rooting recalcitrant species (Le Roux and Van Staden, 1991; Fett-Neto et al., 2001), sometimes making adventitious root (AR) development and vegetative propagation difficult. AR development is a multifactorial process affected by phytohormones, mineral nutrition, genetic traits, environmental factors, stress conditions and plant age, being extremely relevant as the basis for clonal multiplication (Da Costa et al., 2013).

Adventitious roots may develop from several tissues and cell types, including hypocotyl pericycle, vascular parenchyma, young secondary phloem, and interfascicular cambium. AR often emerge in organs such as stems and leaves, but may also derive from non-pericycle tissues in older roots. AR may form as a result of regular developmental programs or in response to several stimuli, such as wounding, stress or hormone applications (Bellini et al., 2014; Druege et al., 2016). Although the early stages of root ontogeny have not been well-described yet, AR induction is characterized by initial biochemical and molecular changes, including high auxin concentration and establishment of a sink for carbohydrates at the wound zone. AR initiation starts with the first visible cell divisions. AR expression involves the growth of root primordia through the stem tissues and establishment of vascular connections. Anatomically, AR development starts with the induction phase, that involves the reprograming of target cells to new meristematic cells (root meristemoids) but absence of any visible cell divisions leading to the formation of internal root meristems. Induction phase is followed by the formation of dome-shaped root primordia (initiation phase), establishment of vascular connections and root emergence (expression phase) (Da Costa et al., 2013; Guan et al., 2015; Druege et al., 2016).

Auxin is a key phytohormone in AR, required in high concentration before root meristemoid formation, and at relatively low concentration for root elongation since it acts as a stimulant during the first stages of AR development and inhibits later developmental stages (Verstraeten et al., 2014). In *Arabidopsis*, flavin-containing monooxygenases (YUCCA) and TRYPTOPHAN AMINOTRANSFERASE OF ARABIDOPSIS (TAA) synthesize indole-3-acetic acid (IAA) from tryptophan. TAA converts tryptophan to indole-3-pyruvate (IPA), whereas YUC produces IAA from IPA (Won et al., 2011). Auxin degradation may be carried out by peroxidases after cutting severance and reduced by antioxidants such as flavonoids, which may also inhibit auxin transport (Da Costa et al., 2013). Shoot tip produced auxin is transported basipetally in the stem by a set of transporters, which include auxin influx AUXIN1/LIKE-AUX1 (AUX/LAX) and asymmetrically distributed efflux PIN-FORMED (PIN) proteins (Guan et al., 2015). Inside the cell, auxin induces expression of auxin-regulated genes by selective proteolysis of its repressors AUXIN/INDOLE-3-ACETIC ACID (Aux/IAA), which act together with the co-repressor TOPLESS (TPL); this releases AUXIN RESPONSE FACTORS (ARFs), which regulate auxin responsive genes (Li et al., 2016). The degradation of Aux/IAA can be mediated by the F-BOX protein TRANSPORT INHIBITOR RESPONSE 1 (TIR1) which is an auxin receptor (Dharmasiri et al., 2005), member of a gene family that includes AUXIN SIGNALING F-BOX (AFBs). Another putative auxin receptor is AUXIN BINDING PROTEIN 1 (ABP1), an extracellular receptor inducing fast responses, especially non-transcriptional (Grones et al., 2015). Auxin also mediates development of AR in response to environmental signals via activation of the WUSCHEL-RELATED HOMEOBOX11 transcription factor (WOX11) in *Arabidopsis*, which is a potential molecular marker to discriminate between adventitious and lateral rooting formation (Liu et al., 2014; Sheng et al., 2017).

Other phytohormones interact with auxin in AR development. Ethylene has been shown to promote early induction and late formation (expression phase) of ARs but to potentially inhibit the late induction phase (Verstraeten et al., 2014). Ethylene action is often strongly interlinked with that of auxin (Da Costa et al., 2013). Many ethylene responsive transcription factors (ERFs) were continuously up-regulated during AR formation (Druege et al., 2016). In *Populus*, members of the transcription factor family APETALA2/ETHYLENE RESPONSE FACTORS (AP2/ERF) had a positive effect on lateral and adventitious rooting (Trupiano et al., 2013). Cytokinins negatively affect adventitious rooting through TYPE B ARABIDOPSIS RESPONSE REGULATORS (ARR) in *Arabidopsis* (Argyros et al., 2008), although the role of these phytohormones appear to be variable. Cytokinins display signaling overlap with auxins, and AR induction is usually promoted by high auxin and low cytokinin levels (Rasmussen et al., 2015). Despite their inhibitory effect on AR induction, cytokinins can have a promotive effect during the first 24 h, when they help drive cell cycle activation (Da Costa et al., 2013).

Clonal propagation of elite genotypes of forest trees is often limited by the loss of rooting competence (Bellini et al., 2014). In *Eucalyptus*, hard-to-root condition has been shown to be associated with low auxin concentration in microcuttings and higher expression of repressors of auxin-responsive genes *TPL*, *IAA12*, and *ARR1*. Exogenous auxin treatment reduced the expression of *TPL* and *ARR1* in *E. globulus*, improving AR development (De Almeida et al., 2015). Far-red light enrichment of donor plants grown in sugar free medium improved rooting of derived microcuttings, correlating with increased allocation of carbohydrates to their basal portion (Ruedell et al., 2013). Simultaneously, higher expression of carbohydrate metabolism-related genes, such as *STARCH SYNTHASE 3* (*SS3*), *SUCROSE-PROTON SYMPORTER 5* (*SUC5*) and *SUCROSE SYNTHASE 1* (*SUS1*) was observed. *SS3* transcripts increased in both donor plants and microcuttings, whereas *SUC5* and *SUS1* augmented in microcuttings. Positive auxin response factors *ARF6* and *ARF8* were also more expressed in donor plants and microcuttings under this condition (Ruedell et al., 2015).

However, little is known about the changes associated with donor plant aging that result in lower rooting capacity, particularly in trees. Guan et al. (2015) suggested that differential auxin biosynthesis and perception could be responsible for easier rooting of juvenile cuttings versus mature and woody ones. We hypothesize that this may also be the case of age-related loss of rooting competence in *E. globulus*. In order to test this assumption, we combined biochemical and molecular tools to examine auxin-related changes associated with loss of rooting capacity in donor plants of *E. globulus* as they age.

## Materials and Methods

### Plant Material

Seeds of *E. globulus* Labill. used in the experiments were pooled from several different non-clonal trees representing the typical rooting recalcitrant phenotype of the species (kindly provided by CMPC Celulose Riograndense S.A., Barra do Ribeiro, RS, Brazil). Seeds were surface-sterilized in 70% ethanol (v/v) for 1 min and 1.5% NaClO (v/v) for 20 min with a few drops of neutral detergent with constant stirring, followed by four washes in sterile distilled water. Groups of fifteen seeds were planted on 300 ml glass jars containing 60 ml of solid culture medium, consisting of 0.5X MS salts (Murashige and Skoog, 1962), 2% sucrose, 0.6% agar, pH adjusted to 5.8 (Fett-Neto et al., 2001), capped with a double layer of aluminum foil. Medium was autoclaved at 121°C for 20 min. The plants were kept under controlled conditions of light and temperature, with photoperiod of 16 h, 40.7 μmol m^-2^ s^-1^ light intensity (provided by white fluorescent lamps) and temperature of 24 ± 2°C. After 30, 45, 60, 75, 90 and 105 days, apical microcuttings were obtained, which were used in morphological, biochemical, and molecular analysis. Samples for the last two purposes were quick frozen in liquid nitrogen and stored at -80°C.

### Morphological Analyses

Microcuttings were transferred and maintained for 20 days in *in vitro* rooting culture medium (devoid of exogenous auxin) containing 0.3X MS salts, 0.4 mg l^-1^ thiamine, 100 mg l^-1^ myo-inositol, 3% sucrose, 0.6% agar (w/v) and pH 5.8 (De Almeida et al., 2010). Medium was autoclaved at 121°C for 20 min. Morphological rooting parameters were analyzed as previously described (De Almeida et al., 2015). Experiments were carried out in 20 ml vials containing 6 ml of medium, capped with a double layer of aluminum foil, at a density of one explant per vial. Two to four independent experiments with 20 explants each were used for determining rooting percentage, length of the longest root per rooted cutting, root number per rooted cutting and mean rooting time. Based on the morphological analyses, the range of post-sowing ages corresponding to rooting competence and loss of this feature were determined and used for guiding harvesting dates in the biochemical and gene expression evaluations. Number of sampling days for each monitored biochemical and molecular variable were chosen based on a series of preliminary assays to estimate relevant time ranges for obtaining both accurate and representative response profiles. Respecting the general parameter of phenotype as a major criterion, times of sample harvest (days after sowing) were as follows: phytohormone-related genes qPCR (45, 75, 105), carbohydrate-related genes qPCR (30, 45, 60, 75), IAA concentration and peroxidase activity (30, 60).

### Total Soluble Sugars Concentration

The extraction of soluble sugars was done according to DuBois et al. (1956) and Ruedell et al. (2013), with minor modifications. Frozen samples of about 15–30 mg of fresh weight (FW) were homogenized in liquid nitrogen, extracted with 750 μl of 80% ethanol (v/v) and incubated in a water bath at 75°C for 15 min. The extracts were centrifuged at 13,000 × *g* for 15 min and the supernatant was recovered. The pellets were re-extracted with 750 μl of 80% ethanol (v/v). The quantification of soluble sugars was done according to McCready et al. (1950), with minor modifications. One hundred μl of the extract was mixed with 600 μl of freshly prepared anthrone reagent [1 g of anthrone in 500 ml of 72% sulfuric acid (v/v)]. The resulting solution was mixed and kept in a boiling water bath for 11 min. After cooling, the absorbance at 630 nm was determined in an M2 Spectramax automated spectrophotometer (Molecular Devices, United States). The standard curve was established with D-glucose.

### Starch Content

To quantify starch content, pellets obtained from the soluble sugars extraction were used (McCready et al., 1950). Pellets were resuspended with 250 μl of distilled water and 320 μl of 52% perchloric acid (v/v), submitted to sonication in a water bath for 15 min and centrifuged at 13,000 × *g* for 15 min. Extraction was done twice. Quantification of starch was as described above for soluble sugars. The standard curve was established with D-glucose in 36.5% perchloric acid (v/v).

### Flavonoid Content

Flavonoid content was essentially determined by the aluminum chloride spectrophotometric method (Zhishen et al., 1999). Approximately 40 mg of frozen plant tissues were ground in liquid nitrogen, extracted in 300 μl 95% ethanol (v/v), and submitted to sonication in a water bath for 30 min in the dark at 4°C. All of the following procedures were carried out under indirect light. The extracts were centrifuged at 12,000 × *g* for 10 min at 4°C and the supernatants were collected. For quantification, 100 μl of extract was added to 400 μl of water and 30 μl of 5% NaNO_2_ (w/v), mixed and then kept at 25°C for 5 min. Next, 30 μl of 1% AlCl_3_ (v/v) was added, mixed and then incubated at 25°C for 6 min. Then, 200 μl of 1 M NaOH and 240 μl of water were added and mixed. Reading was done at 510 nm in spectrophotometer. The standard curve was established with quercetin (Sigma, St. Louis, MO, United States).

### Gene Expression

Total RNA was isolated from tip cuttings of 30, 45, 60, 75, and 105-day-old donor plants using NucleoSpin^®^ RNA Plant Kit (Macherey-Nagel), including DNAse I treatment, following the manufacturer recommendations. *ERF* (Eucgr.C02719),*ARR1* (Eucgr.A00189.1), *TPL* (Eucgr.C01368.1), *IAA12* (Eucgr.H02914.1), *ABP1* (Eucgr.H01544.1), *TIR1* (Eucgr.K03439.1), *AUX1* (Eucgr.A00514.1), and *PIN1* (Eucgr.K02271.1) were examined at 45, 75, and 105 days after sowing, whereas *TAA1* (Eucgr.H02519.1), *YUC3* (Eucgr.H02874.1), *SS3* (Eucgr.D01324) and *SUS1* (Eucgr.C03199), at 30, 45, 60, and 75 days. Genes were selected on the basis of their involvement in rooting responses of *E. globulus* indicated by other studies focusing on microcuttings (De Almeida et al., 2015; Ruedell et al., 2015). Sequences of *Arabidopsis* genes were used as query for searching the respective orthologs in *Eucalyptus*, except for *ARR1* whose search was performed using the *PtRR13* sequence from *Populus* as query, an ortholog of the *Arabidopsis ARR1* (De Almeida et al., 2015). Total RNA concentration was determined using a Nanodrop^®^ Spectrophotometer (Thermo Scientific), whereas RNA quality was monitored by electrophoresis in 1% agarose gel. One independent cDNA synthesis was done for each sample and first strand cDNA synthesis was carried out using 100 ng of total RNA, oligo-dT primers and reverse transcriptase M-MLV (Invitrogen^TM^) in a final volume of 20 μl. The final cDNA products were diluted 100 fold in RNAse free Milli-Q^®^ water prior to use in qPCR.

All qPCR analyses were conducted with fast optical 48 well-reaction Plates 0.1 ml (MicroAmp^TM^ Applied Biosystems) using Platinum Taq DNA polymerase (Invitrogen^TM^) and SYBR Green dye in a StepOne^TM^ Real-Time PCR System (Applied Biosystems), according to manufacturer instructions. Reactions were kept at 95°C for 5 min, followed by 40 cycles of 95°C for 15 s, 60°C for 10 s, and 72°C for 15 s. PCR specificity was checked by heat dissociation curve from 60 to 90°C, following the final PCR cycle. Reactions were carried out in technical quadruplicates for each cDNA sample and biological triplicates. Primers for analyzed genes were designed with Primer 3 software (Koressaar and Remm, 2007; Untergasser et al., 2012), as described in De Almeida et al. (2015) and Ruedell et al. (2015). Reference genes were *HISTONE H2B* (*H2B*) and *ALPHA TUBULIN* (*TUA*) (De Almeida et al., 2010). The majority of these genes had their function established in *Arabidopsis* or other plant species, so for *E. globulus* they are in fact ‘like’ genes. For the sake of easy format of the text, however, they will be referred to by gene names as found in the databases. Data analyses were done by Comparative Ct method (Pfaffl, 2001), whereas relative expression was measured using each reference gene separately, followed by calculation of the respective mean relative expression.

### Total Peroxidase Activity and Protein Content

Approximately 50 mg of frozen plant tissue were ground in liquid nitrogen and total protein content was extracted in 998.75 μl of 0.2 M phosphate buffer pH 7.0 plus 1.25 μl of Protease Inhibitor Cocktail (Sigma, United States) and 5 mg of soluble polyvinylpolypyrrolidone (PVP) – added to buffer at least 10 h before extraction-mixed, centrifuged at 13,000 × *g* for 20 min at 4°C and supernatant was collected. Peroxidase specific activity was quantified according to Fett-Neto et al. (1992), with some modifications. Enzyme activity was assayed in a mixture containing: 1.5 ml of 0.2 M phosphate buffer pH 7.0, 250 μl of 1% H_2_O_2_, 250 μl of 1% guaiacol and 75 μl of enzyme extract. The phosphate buffer replaced the extract in the blank. The reaction was measured by the change in absorbance at 420 nm. Measurements were taken with a spectrophotometer every 30 s for 9 min. The initial linear part of the curve was used to determine peroxidase specific activity, which was expressed by change in absorbance at 420 nm min^-1^ mg^-1^ (protein), at 25 ± 2°C under diffuse light. Crude protein extracts in 0.2 M phosphate buffer pH 7.0 were used to determine protein concentration. Protein was quantified by the method of Bradford (1976) and the standard curve was established with bovine serum albumin (BSA).

### Endogenous Auxin Quantification by HPLC

Endogenous auxin extraction and quantification from tip cuttings of plants with 30 and 60 days after sowing were performed according to Kim et al. (2006), with modifications as described by De Almeida et al. (2015). Briefly, chromatography was carried out on a Shimadzu C18 reverse-phase column (250 mm × 4.6 mm), with corresponding guard column, in a Shimadzu SPD-20A HPLC equipment using a gradient system of three mobile phases: Solvent A: 10% methanol, 0.3% acetic acid; Solvent B: 90% methanol, 0.3% acetic acid; Solvent C: 100% acetonitrile. All solutions were previously filtered through 0.45 μm Millipore^®^ membranes and degassed. Flow rate was 1.0 ml min^-1^ and detection was done with a Shimadzu RF-10A XL fluorescence detector (Emission at 360 nm, Excitation at 282 nm). To quantify IAA, 20 μl of each sample was injected, and an external standard curve was generated using IAA (Acros Organics, Geel, Belgium). The identification of IAA content from samples of *E. globulus* was based on retention time and co-chromatography with authentic IAA standard. The contents of IAA in samples were expressed as nmol of IAA per gram of extracted fresh weight.

### Experimental Layout and Statistics

All assays herein described were performed in totally randomized layout, with biological triplicates or quadruplicates. The results were analyzed by ANOVA followed by Tukey test or Student’s *t*-test, whenever appropriate, using the statistic packages GraphPad Prism 6 and GraphPad QuickCalcs: *t-*test calculator. *P* ≤ 0.05 was used in all cases.

## Results

### Donor Plant Aging Negatively Affects Morphological Rooting Parameters

**FIGURE 1:**
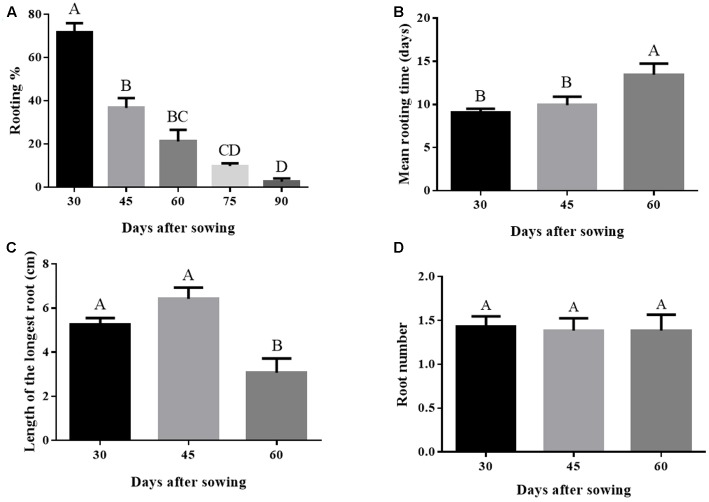
**(A–D)** Rooting performance in *Eucalyptus globulus* microcuttings derived from donor plants of different ages after sowing. Bars not sharing a letter are significantly different by Tukey test (*P* ≤ 0.05). Values are the means of 2 to 4 independent experiments. Data on graphs **(C)** and **(D)** considered only rooted cuttings. Lines on top of bars represent standard errors.

Adventitious root development capacity declined with time. Rooting percentage is 35 fold higher on day 30 compared with day 90 (**Figure 1A**). Aging also negatively affected the mean rooting time, wherein the 60 day old microcuttings developed the first visible root only after 13 days of culture (**Figure 1B**). Length of the longest root also decreased significantly in microcuttings derived from 60-day-old donor plants. Longest root length decreased from 5.2 (30-day-old donor plants) to 3.1 cm (60-day-old donor plants) (**Figure 1C**). On the other hand, no significant effect on root number was observed (**Figure 1D**).

### Age-Related Changes in Carbohydrate, Flavonoids, and Protein Concentrations

The concentrations of soluble carbohydrates, starch, total flavonoids, and soluble protein in tip cuttings of donor plants with different ages did not show significant changes between 30 and 75 days of age (Supplementary Table S1). A slight increase in starch concentration over time could be observed.

### Gene Expression

**FIGURE 2:**
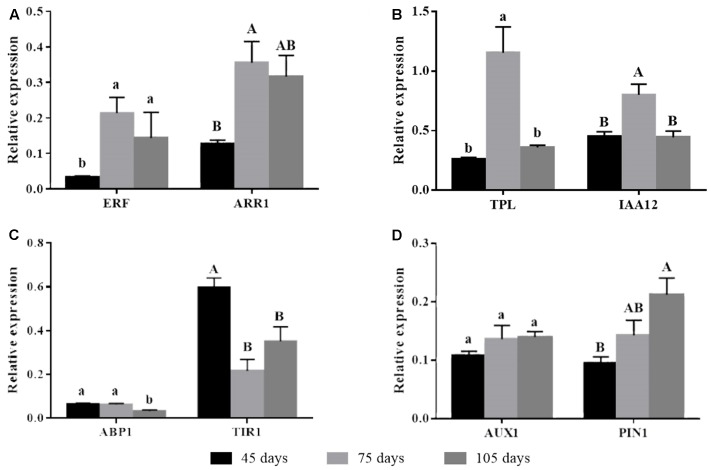
Gene expression of auxin action mechanism-related genes in *E. globulus* tip cuttings as a function of donor plant age (rooting competence diminishes between 45 and 75 days). Black bars – 45 days; light gray bars – 75 days, dark gray bars – 105 days. **(A)** Ethylene and cytokinin auxin crosstalk-related genes *ERF* and *ARR1*. **(B)** Inhibitors of auxin responsive genes *TPL* and *IAA12*. **(C)** Auxin receptors *ABP1* and *TIR1*. **(D)** Auxin transporter genes *AUX1* and *PIN1*. Expression levels genes were evaluated using qPCR and normalized against *H2B* and *TUA* as endogenous control genes. Values represent average of biological triplicates analyzed in technical quadruplicates. Bars sharing the same letters are not different according to Tukey test with *P* ≤ 0.05. Lines on top of bars represent standard errors.

Auxin receptor genes had differential expression over time. Expression of *TIR1* was higher on day 45 and diminished on subsequent times. *ABP1* showed lower expression compared to *TIR1* and also had its lowest expression level on day 105 (**Figure 2C**). The auxin action repressor genes *TPL* and *IAA12* had increased expression on day 75, going back to the levels observed on day 45 after 105 days (**Figure 2B**). A similar expression profile of the time corresponding to rooting phenotype transition was observed for cytokinin-related *ARR1* (**Figure 2A**). The decreased expression of the auxin receptor *TIR1* and the concomitant increased expression of the auxin repressors *TPL*, *IAA12*, and *ARR1* coincided with the loss of rooting capacity in microcuttings derived from the donor plants, which occurred between days 45 and 75 (**Figure 1A**). However, the expression of the root promoting ethylene-related transcription factor *ERF* increased as rooting capacity reduced (**Figure 2A**). Expression of auxin transporters was not well-correlated with loss of rooting capacity. *AUX1* showed stable expression, while *PIN1* had a significant increase in expression only at day 105 (**Figure 2D**).

**FIGURE 3:**
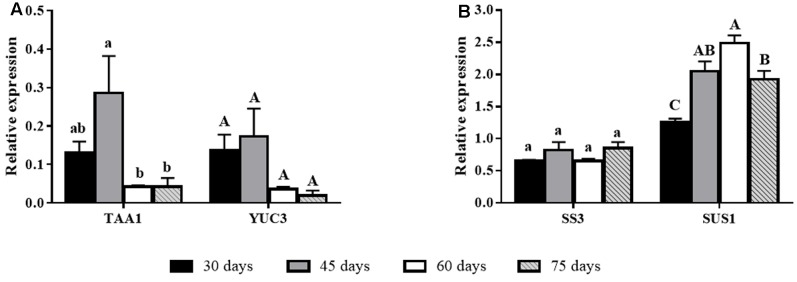
Gene expression of biosynthesis-related genes in *E. globulus* tip cuttings as a function of donor plant age (rooting competence diminishes between 30 and 60 days). Black bars – 30 days, gray bars – 45 days, white bars – 60 days, diagonal line bars – 75 days. **(A)** Auxin biosynthesis genes *TAA1* and *YUC3* during aging in *E. globulus*. **(B)** Carbohydrate biosynthesis genes *SS3* and *SUS1* during aging in *E. globulus*. Expression levels genes were evaluated using qPCR and normalized against *H2B* and *TUA* as endogenous control genes. Values represent average of biological triplicates analyzed in technical quadruplicates. Bars sharing the same letters are not different according to Tukey test with *P* ≤ 0.05. Lines on top of bars represent standard errors.

Biosynthetic related genes had variable expression as donor plants aged. The auxin biosynthetic gene *TAA1* had higher expression in younger plants (high rooting competence), whereas *YUC3* showed a similar trend, albeit not statistically significant (**Figure 3A**). Expression of carbohydrate biosynthesis genes was not clearly correlated to rooting ability. *SS3* had stable levels of expression, whereas *SUS1* showed lower expression at day 30, stabilizing at higher levels after 45 days (**Figure 3B**).

### Peroxidase Activity and Auxin Concentration

**FIGURE 4:**
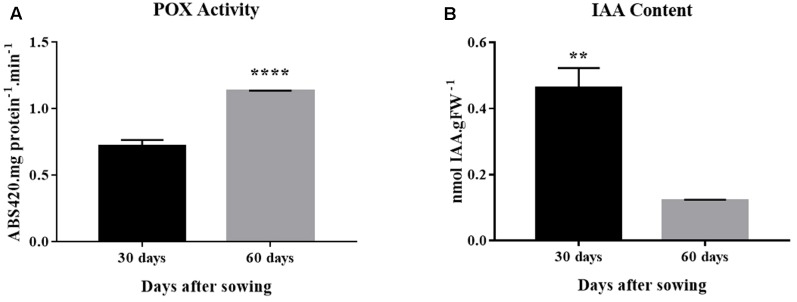
Peroxidase activity and auxin concentration in *E. globulus* tip cuttings as a function of donor plant age (rooting competence diminishes between 30 and 60 days). **(A)** Peroxidase (POX) activity. **(B)** Endogenous indol-3-acetic acid (IAA) concentration. Asterisks indicate significant difference according to Student’s *t*-test: ^∗∗^*P* ≤ 0.01 and ^∗∗∗∗^*P* ≤ 0.0001. Lines on top of bars represent standard errors.

Peroxidase activity increased by approximately twofold from 30 to 60 days of age, a time window within which rooting competence reduced significantly (**Figures 1A, 4A**). The profile observed for IAA concentration was exactly the opposite. Auxin concentration decreased 75% between 30 and 60 days (**Figure 4B**).

## Discussion

The aseptic cultivation system used in this work provides a means to accelerate the progress of decline in rooting capacity of microcuttings from donor plants, so it is manifested at a very young age. The culture protocol probably provided a set of conditions leading to physiological aging that impacted rooting ability (Leakey, 2014). Progressive loss of adventitious rooting capacity of microcuttings derived from aging donor plants was evident, becoming significant around 1.5 to 2 months after sowing. This can be inferred by taking together several rooting parameters, such as percent rooting, mean rooting time and longest root length. By comparing profiles before and after the decline of adventitious rooting ability, biochemical and gene expression of donor plants provided clues about the underlying mechanisms.

The absence of differences in the concentration of proteins, flavonoids, starch, and soluble carbohydrates suggests that these parameters were not major drivers of the changes in rooting competence of explants from donor plants as they age. Flavonoids may affect AR development as auxin transport inhibitors or as antioxidants that may protect auxin pools from degradation, whereas carbohydrates are essential for energy metabolism and as biosynthetic units, thereby playing a central role in root development (Da Costa et al., 2013).

Studies with *Eucalyptus* have shown a regulatory role for carbohydrates in AR development (Corrêa et al., 2005). It is possible that flavonoid and carbohydrate metabolic changes after cutting severance may contribute to the different rooting outcomes as a function of donor plant age. Gene expression data showed no changes in *SS3* and an increase in *SUS1* as rooting competence decreased. Since plant SUS1 is mostly a sucrose degrading enzyme (Ruan, 2014), this may indicate an increase in sink strength or carbon relocation toward the site of rooting (Ruedell et al., 2015), although total carbohydrate concentrations were not significantly changed. This profile could presumably be beneficial to rooting, albeit several other factors could override it, resulting in the recalcitrant phenotype of aged donor plants. Protein content is relevant for both structural and regulatory/metabolic processes, and it is closely dependent on the availability of nitrogen sources. The stable concentration of proteins indicated that nitrogen supply was adequate throughout the experiments.

Auxin sensitivity loss may occur due to lower expression of the main auxin receptor *TIR1* as donor plants age. After microcuttings are severed, basipetally transported auxin from shoot tips and young leaves accumulates at the cutting base, creating a local concentration gradient that drives the induction of AR (Da Costa et al., 2013; Druege et al., 2016). In older donor plants, reduced expression of the auxin receptor may increase the response threshold, partially impairing rooting. Based on expression data, however, auxin transport was maintained relatively stable during the rooting loss transition, with a significant increase in *PIN1* transcript level at a much later time point (105 days). Apparently, the putative increased transport did not overcome sensitivity decrease, which was also coupled with lower auxin concentration in cuttings and increased peroxidase activity, some isoforms of which may be capable of oxidizing this phytohormone (Peer et al., 2013).

The drop in IAA steady-state concentration coincident with lower rooting capacity may have resulted from reduced biosynthesis, increased conjugation or degradation. The relationship between lower concentration of IAA and increased total peroxidase activity as rooting competence is lost must be viewed with caution. Plant peroxidases are encoded by a large gene family, as described for *Eucalyptus grandis* (Li et al., 2015), isoforms may have different subcellular locations and substrate specificities. Besides, compelling evidence of *in vivo* activity of peroxidase as a major factor regulating auxin homeostasis in intact plants is lacking. In fact, some studies indicate that these enzymes would play a minor role compared to peroxidase-independent degradation (Peer et al., 2013). In cuttings, total peroxidase activity has been considered a biochemical marker of AR development (Fett-Neto et al., 1992; Schwambach et al., 2008), and the present study suggests this may also be the case for rooting competence of donor plants of *E. globulus*, at least under the experimental conditions described herein. On the other hand, expression of the biosynthetic gene *TAA1* suggested that there is diminished synthesis of auxin at time points when rooting competence is significantly lower, a trend that is also followed by *YUC3* (significant at *P* = 0.08), which acts in the same pathway (Won et al., 2011). A comparison of different *E. globulus* clones showed that basal endogenous IAA concentration of hard-to-root microcuttings was significantly lower than that of easy-to-root ones (Negishi et al., 2014). A change in auxin homeostasis involving a decrease in the IAA pool was found to correlate with vegetative to floral switch in pea and concomitant decline in AR development (Rasmussen et al., 2015). Interestingly, we observed that differences in gene expression patterns and auxin metabolic homeostasis of eucalypt donor plant tip shoots prior to and after rooting competence loss are similar to those of easy (*E. grandis*) and hard (*E. globulus*) -to-root microcuttings during rhizogenesis (De Almeida et al., 2015).

Auxin is also known for regulating different downstream factors that can affect AR development, such as nitric oxide and microtubule-related transcripts (Steffens and Rasmussen, 2016; De Almeida et al., 2017). Juvenile cuttings derived from 6-month old plants of *E. grandis* showed higher nitric oxide production (Abu-Abied et al., 2012) and increased responsiveness of microtubule-related transcripts upon auxin treatment (Abu-Abied et al., 2014) compared to their mature counterparts. Although not examined, these downstream events may be at play in the experimental model using *E. globulus* seedlings described herein.

Auxin signaling-related genes with major functional roles showed expression profiles that varied as donor plants aged and lost rooting competence. The auxin action repressor genes *TPL* and *IAA12* displayed higher expression at the transition from full root competence to reduced competence, later returning to initial levels. The peak of expression of these repressor genes is consistent with a decrease in auxin action, thereby compromising AR development. The similar expression kinetics makes functional sense, since TPL directly interacts with the EAR domain on IAA12/BDL for repressing auxin response genes (Szemenyei et al., 2008). The subsequent return of *TPL* and *IAA12* to initial levels of expression could be a response to the reduced auxin concentration and sensitivity (based on *TIR1* expression). The prominent role of auxin activity and homeostasis in the loss of rooting capacity concurs with the fact that exogenous supply of auxin to microcuttings derived from 3-month-old stock plants restores their rooting ability (Fett-Neto et al., 2001).

Adventitious root development depends on the input of several phytohormones besides auxin. Among these, cytokinins and ethylene play significant roles primarily as rooting inhibitors and stimulators, respectively (Druege et al., 2016). In *Arabidopsis*, cytokinin regulates auxin action negatively through upregulation of *AUX/IAA* as well as reduction in auxin flow by restriction in PIN1 and LAX3 expression domains, increasing rooting recalcitrance (Della Rovere et al., 2013). The poplar *PtRR13* cytokinin type B response regulator inhibits AR development (Ramírez-Carvajal et al., 2009). Rooting of *E. globulus* microcuttings was also inhibited by cytokinin exposure (Corrêa et al., 2005). In the cambium region of *E. globulus* microcuttings, the cytokinin regulator type B *ARR1* is more expressed compared to the same tissues of the easy-to-root *E. grandis*. Besides, the same gene is downregulated by auxin treatment, leading to improved AR development (De Almeida et al., 2015). In good agreement, the expression of *ARR1* increased as rooting competence decreased, suggesting a change in the balance of root promoting auxins versus root inhibiting cytokinins.

In contrast, *ERF*, member of a family of transcription factors known as positive regulators of lateral and AR development (Trupiano et al., 2013; Druege et al., 2016), showed increased expression associated with loss of rooting ability (**Figure 2A**). Since ERFs are responsive to ethylene, this may be a function of ethylene accumulation as donor plants age in the culture vessels. Adventitious rooting in microcuttings of *E. globulus* and *Eucalyptus saligna* was not strongly influenced by silver nitrate – an ethylene inhibitor (Fogaça and Fett-Neto, 2005), whereas, in hybrid *Eucalyptus*, ethylene has been shown to inhibit cutting adventitious rooting, since the use of two different ethylene inhibitors improved rooting (Kilkenny et al., 2012). It is possible that some of these contradictory results may reflect differences in ethylene concentrations and/or species characteristics. On the other hand, AP2/ERFs transcription factors form a large family, involved in regulating plant responses to various types of stresses, not necessarily related to AR development (Phukan et al., 2017).

## Conclusion

**FIGURE 5:**
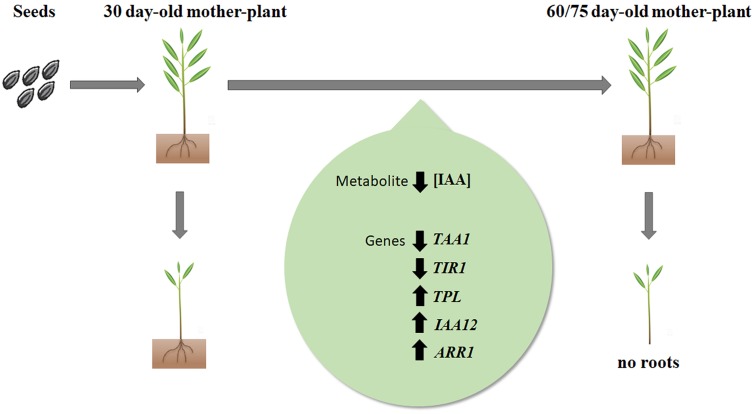
Working model of biochemical and molecular changes associated with loss of adventitious rooting capacity in *E. globulus* Labill. microcuttings as a function of mother-plant age. Decrease in concentration of endogenous IAA as well *TAA1* and *TIR1* expression, associated with increase in expression of *TPL, IAA12* and *ARR1*, lead to loss of rooting capacity. Upward arrows represent increased gene expression; downward arrows represent decreased gene expression or IAA concentration.

Taken together results point to a multifactorial mechanism leading to significant loss of rooting competence of microcuttings derived from aged donor plants of *E. globulus*. A tentative summarizing model is proposed (**Figure 5**). As rooting competence decreases, the main factors at play appear to be a reduced content and biosynthesis of IAA (*TAA1*), reduced sensitivity to auxin (*TIR1*), and increased activity of auxin action inhibitors (*TPL, IAA12*), including transcription factors involved in cytokinin-auxin crosstalk (*ARR1*). These data may provide insights on how to overcome or delay loss of rooting competence in order to rescue propagation effectiveness of industrially relevant *E. globulus* genotypes.

## Author Contributions

MA was involved in experimental design and overall execution, as well as drafting the manuscript. MdA helped in tissue culture assays and gene expression experiments. AdA worked in qPCR experiments. HM assisted in the biochemical assays, CdOJ helped in overall execution and statistical analysis and FdC in HPLC assays for IAA quantification. AF-N conceived and supervised the work and finalized the manuscript.

## Conflict of Interest Statement

The authors declare that the research was conducted in the absence of any commercial or financial relationships that could be construed as a potential conflict of interest.
