# Supplementary material for: Events Associated with Early Age-Related Decline in Adventitious Rooting Competence of Eucalyptus globulus Labill
Source: Front Plant Sci. 2017 Oct 10;8:1734. doi: 10.3389/fpls.2017.01734 (PMC5641372; doi:10.3389/fpls.2017.01734)
Supplement: Supplementary file 1 [file Table_1.DOCX]

Supplementary Material

**Events associated with early age-related decline in adventitious rooting competence of *Eucalyptus globulus* Labill**

**Márcio Luís Aumond Júnior, Artur Teixeira de Araujo Júnior, Camila Fernanda de Oliveira Junkes, Márcia Rodrigues de Almeida, Hélio Nitta Matsuura, Fernanda de Costa and Arthur Germano Fett-Neto^*^**

*** Correspondence:** Corresponding Author: Dr. Arthur Germano Fett-Neto, [fettneto@cbiot.ufrgs.br](https://mailserver.cbiot.ufrgs.br/horde/imp/message.php?mailbox=INBOX&index=50098)

# Supplementary material

**Table S1.** Concentrations of starch, soluble sugars, flavonoids, and proteins (means ± standard errors) in just harvested tip microcuttings obtained from donor plants of different ages after sowing. Data are the means of two to three independent experiments with biological triplicates. There is no significant difference among data derived from microcuttings (Tukey test, P ≤ 0.05). DW= dry weight, FW=fresh weight

|  |  |  | Flavonoids |  |
| --- | --- | --- | --- | --- |
|  | Starch | Soluble sugars | Quercetin equivalents | Protein |
|  | (mg.mg^-1^ DW) | (mg.mg^-1^ DW) | (µg.mg^-1^ DW) | (mg.mg^-1^ FW) |
| Days after sowing |  |  |  |  |
| 30 | 31.83 ± 2.911 | 81.13 ± 7.777 | 7.569 ± 0.479 | 13.53 ± 0.597 |
| 45 | 34.41 ± 3.877 | 85.98 ± 9.585 | 7.345 ± 0.496 | 13.41 ± 0.515 |
| 60 | 37.36 ± 3.822 | 92.72 ± 10.430 | 6.156 ± 0.457 | 13.82 ±0.382 |
| 75 | 41.47 ± 2.979 | 91.02 ± 9.156 | 6.931 ± 0.509 | 13.00 ± 0.357 |
